# Burnout and perceived stress in medical interns working at a hospital in Windhoek, Namibia

**DOI:** 10.4102/jcmsa.v4i1.302

**Published:** 2026-03-10

**Authors:** Frieda Kalenga, Liezl Koen, Laila Asmal, Moleen Zunza, Roxane Jones

**Affiliations:** 1 Department of Psychiatry, Faculty of Medicine and Health Sciences, Stellenbosch University, Cape Town, South Africa; 2 Division of Epidemiology and Biostatistics, Faculty of Medicine and Health Sciences, Stellenbosch University, Cape Town, South Africa

**Keywords:** burnout, perceived stress, medical interns, Namibia, hobbies

## Abstract

**Background:**

Namibian medical interns face challenges that may contribute to financial strain, growing dissatisfaction and protests over deteriorating working conditions, which may increase vulnerability to perceived stress and burnout. This study aimed to investigate the prevalence of burnout and perceived stress among medical interns in Namibia and to examine the associations between these two factors and selected demographic characteristics. The study was conducted at the Windhoek Central Hospital and Katutura State Hospital (WCH-KSH) Complex in Windhoek, Namibia.

**Methods:**

A cross-sectional survey, which included a socio-demographic questionnaire, the Copenhagen Burnout Inventory (CBI) and the Perceived Stress Scale (PSS) was conducted between December 2023 and January 2024. Data were analysed using univariable and multivariable logistic regression to identify associations between burnout, perceived stress and demographic factors.

**Results:**

Out of 107 interns who initiated the survey, 76 completed the full survey. The overall burnout prevalence was 79.4%, with personal burnout (96.0%) and work-related burnout (93.6%) being the most common subtypes. High perceived stress was present in 63.8% of participants and was significantly associated with burnout (adjusted odds ratio [aOR] = 1.28, 95% confidence interval [CI]: 1.06–1.62, *p* = 0.02). Engagement in hobbies was associated with a lower odds ratio of burnout (odds ratio [OR] = 0.17, 95% CI: 0.06–0.71, *p* = 0.01).

**Conclusion:**

Burnout and perceived stress measured on the CBI and PSS scales are highly prevalent in this sample of medical interns in Namibia. Workload-related stressors appear to be key contributors, while engagement in hobbies was associated with lower odds of burnout.

**Contribution:**

The findings of this study highlight the need for targeted interventions focused on workload management and access to psychological support in this occupational group.

## Introduction

Burnout has been recognised as a workplace phenomenon for over 20 years, first described by Freudenberger in the 1970s as a state of physical and emotional exhaustion experienced by workers exposed to prolonged occupational stress.^1^ Maslach and Leiter later developed a comprehensive framework, describing burnout as a syndrome encompassing emotional exhaustion, depersonalisation and reduced professional efficacy.^2^ Over the decades, burnout has evolved from being seen primarily as an individual response to chronic stress to a complex construct influenced by organisational, interpersonal and systemic factors.^3,4^ Burnout is prevalent among medical professionals and is a major concern globally. It is characterised by physical and emotional exhaustion from long-term exposure to emotionally demanding work. Burnout affects interpersonal skills, job performance, career satisfaction and psychological health.^3,4^ Consequences include adverse effects on patient care, reduced desire to help and reduced willingness to work.^5,6,7,8^ Dybre et al.^9^ reported that burnout is more prevalent in physicians at all stages of their training than in their peers from the general population.

Global studies have reported high prevalences of burnout, with a systematic review by Rotenstein et al.^10^ reporting rates of burnout at 72%. Studies conducted in South Africa and other African countries have identified a high prevalence of burnout, ranging between 58.9% and 78%.^11,12,13^ Previous studies have found that many medical trainees experience burnout.^11,14^ A study on burnout in Irish medical interns reported a prevalence of 37%,^6^ while a study of Malaysian medical interns reported a prevalence of personal (73.3%) and work-related (69.1%) burnout, with a lower patient-related burnout (43.4%).^14^

Burnout has been studied among Namibian medical doctors, nursing professionals and medical students.^15,16,17^ A study of medical doctors working at a maternity unit at the Windhoek Central Hospital-Katutura State Hospital (WCH-KSH) Complex showed that over half of the participants were at risk or at very high risk of burnout,^13^ while another study reported that more than a third of medical students were found to be depressed or burnt out.^17^ Buitendach and Moola^16^ reported on factors mitigating the development of burnout in nursing staff in the same hospital complex and found that emotional exhaustion and cynicism were associated with lower work engagement and emotion-focused coping acted as a buffer against cynicism and burnout in nurses with lower job satisfaction. However, we are not aware of any study where the relationship between perceived stress and burnout in Namibian medical interns has been investigated.

The internship is a critical point in a medical professional’s journey, reflecting a transition from a student to an independently functioning physician. It is an important milestone in a physician’s life.^4^ It typically involves long working hours, with associated chronic sleep deprivation, which, in turn, has been associated with numerous negative mood changes, memory loss, over-optimistic risk-taking, increased motor vehicle accidents and needle stick injuries with possible exposure to infectious diseases.^18^ High levels of stress are a possible precursor to burnout. Within the context of an internship, this notion is supported by an Ethiopian study, which showed that medical interns who reported high levels of perceived stress had a threefold increased risk for emotional exhaustion.^11^

Socio-demographic factors such as level of education, socioeconomic status, neighbourhood profile and gender, as well as behavioural risk factors such as smoking and alcohol consumption, have been associated with high perceived stress.^19^ The early identification of individuals and subgroups with an accumulation of stress-related risk factors may provide opportunities for early strategic intervention for the prevention of adverse behavioural and health outcomes.^20^

According to the Health Professional Council of Namibia, the regulatory body for healthcare professionals in Namibia, all medical graduates are mandated to complete 2 years’ supervised training in an academic hospital before they can be registered as an independent medical practitioner. In recent years, Namibia’s medical internship programme has faced significant challenges. In addition to the typical demands of a medical internship, Namibian medical interns face challenges about poor working conditions reported in the lay press, including excessive work hours, unilateral reduction in remuneration and the withdrawal of previously provided medical aid.^21^ These systemic issues not only contribute to financial strain but may also have led to reported dissatisfaction and protest over the deteriorating working conditions, which are likely to increase vulnerability to both perceived stress and burnout in this population.

Burnout and work-related stress among doctors have been the focus of much attention recently. Understanding the factors associated with stress during the early career period of internship may help with developing interventions to reduce and prevent stress. This study aims to investigate the prevalence of burnout and the associated perceived stress among medical interns at the WCH-KSH Complex in Windhoek, Namibia and to determine whether any socio-demographic factors are associated with burnout and perceived work stress.

## Research methods and design

### Study design

We conducted a cross-sectional study to determine the prevalence of burnout and perceived stress among medical interns in Namibia between December 2023 and January 2024.

### Setting

This study was conducted at the WCH-KSH Complex in Windhoek, Namibia. These hospitals serve as the only training centres for medical interns in Windhoek. Katutura State Hospital is a general referral hospital that provides a wide range of medical services to the community, with approximately 840 beds. Windhoek Central Hospital is a tertiary national referral hospital, offering specialised medical care across various disciplines with a bed capacity of 855 beds.

### Study population and sampling strategy

All medical interns who were completing their internship at WCH-KSH Complex from December 2023 to January 2024 were eligible for inclusion. The medical interns complete a 2-year training programme, rotating through various specialities. They spend 4 months each in Internal Medicine, Surgery, Obstetrics and Gynaecology, and Paediatrics, and 2 months each in Orthopaedics, Anaesthesia, Psychiatry, and Family Medicine. Some internships may extend beyond 2 years if all the required rotations have not been completed. At the time of the study, 300 medical interns were undergoing training at WCH-KSH Complex. Eligibility to participate was limited to medical interns employed at WCH-KSH Complex at the time of the study.

We estimated the required sample size for this study using the prevalence of different types of burnout as reported by Roslan et al.^14^ using the WINPEPI software.^22^ The lowest reported prevalence, client-related burnout at 43%, yielded a sample size of 161 participants, with an estimated margin of error of ±7.65%. A voluntary response sampling strategy was used to recruit participants.

### Data collection

The study population were invited to complete an anonymous online self-report survey administered using REDCap. To maintain confidentiality, the intern representatives distributed the survey link through existing official social media platforms to ensure that no personal information (such as names or email addresses) was accessible to the research team.

The study was conducted in English only, and participation was voluntary. The WCH-KSH Complex’s intern curator and intern representatives shared information about the study during departmental meetings and distributed the link to complete the online survey. The survey took approximately 30 min to complete. Three email reminders were sent every 2 weeks to remind interns of the survey. To encourage participation, participants were offered the opportunity to win one of three R500 gift vouchers in a lucky draw. The survey was shared with over 300 medical interns; 107 (36%) completed the socio-demographic questionnaire, 97 (32%) completed up to the end of the PSS tool, and 78 (26%) participants completed the whole survey. The number of participants included in each analysis (*n*) is explicitly reported in the results section.

### Measures

Participants completed three self-report questionnaires: firstly, a socio-demographic questionnaire that was developed by the research team specifically for this study to assess socio-demographic factors as related to burnout; secondly, the Copenhagen Burnout Inventory (CBI) to assess interns’ level of burnout, and thirdly, the Perceived Stress Scale (PSS) to assess the interns’ perceived level of stress.

#### Copenhagen burnout inventory

The CBI is a 19-item self-reported measure of burnout and consists of three subscales: personal burnout, work-related burnout and client-related burnout.^23^ All items are rated on a five-point Likert scale ranging from 1 (never or almost never) to 5 (always). Scores range from 0 to 100 and are calculated for the total scale and each subscale. Overall burnout was calculated as the average of the three subscales.^24^ A burnout threshold score of ≥ 50 was used to classify participants as having burnout for each subscale and for overall burnout.^14,25^ The CBI has shown satisfactory reliability and validity as a tool to measure burnout.^23^ The CBI has not yet been validated in a Namibian population, but has been validated and used in other similar low- to middle-income country (LMIC) populations.^14,26,27^

#### The perceived stress scale

The PSS is a self-appraisal measure for individuals to assess the extent of the perceived stressfulness of their current life situation.^28^ The scale comprises 10 items rated on a five-point Likert scale (0 = never to 4 = very often). Items 4, 5, 7, and 8 are reverse-scored, and the total score is summed. Total scores were obtained by summing all the items; the scores were divided into tertiles to classify stress levels: low perceived stress (1st tertile), moderate perceived stress (2nd tertile) and high perceived stress (3rd tertile).^11,29^ For logistic regression analysis, moderate and high stress categories were combined into a single high stress group. The PSS has been widely used and psychometrically validated as a reliable measure of psychological stress estimated over the preceding 4 weeks.^28^ This tool has not been validated in a Namibian population but has been used and validated in other LMICs.^11,29,30,31^

### Statistical analysis

Data analysis was performed using Stata 18 (College Station, Texas, United Sates). All variables are reported as counts and percentages. The primary outcome was burnout (yes/no), and the secondary outcome was perceived stress (high/low).

Binary logistic regression was used to test associations between burnout and demographic variables (age, gender, marital status, training year, faith participation, hobbies and pet ownership) as independent predictors. Binary logistic regression was used to investigate associations between perceived stress and demographic factors, with perceived stress (high/low) as the dependent variable. Association between burnout and perceived stress was tested using a logistic regression model adjusting for confounding variables, as determined by univariate analysis (variables with *p* < 0.1). Odds ratios (ORs) with 95% confidence intervals (CIs) are reported. A multivariable model was not developed for perceived stress, as perceived stress was conceptualised as a predictor of burnout rather than the primary outcome in this study’s theoretical framework. Statistical significance was set at *p* < 0.05.

### Ethical considerations

Ethics approval for this study was obtained from the Health Research Ethics Committee of Stellenbosch University (Ref no.: S23/07/162). The study was also approved by the Namibian Ministry of Health and Social Services Research Unit (Ref: 22/4/2/3) and the Management of Windhoek Central Hospital and Katutura State Hospital. Participation was voluntary with anonymity maintained throughout, and the consent document stated that implied consent would be indicated by completion of the questionnaires. The study was conducted in accordance with the Declaration of Helsinki^32^ and the Guidelines for Good Clinical Practice.^33^

## Results

### Participant characteristics

A total of 107 (36%) medical interns completed the socio-demographic questionnaire. Most participants were aged 26–34 years (*n* = 87, 81.3%) and identified as female (*n* = 86, 80.4%) (Table 1). Participants were distributed across various clinical rotations, with the largest proportion in Internal Medicine (*n* = 26, 24.3%). Similar numbers were in their first (*n* = 50, 47.2%) or second (*n* = 49, 46.2%) year of training, with a small proportion (*n* = 7, 6.6%) completing an extended internship beyond 2 years. Regarding relationship status, 54.6% (*n* = 58) were single, and in terms of lifestyle factors, just over two-thirds reported active faith participation (*n* = 73, 68.2%), 29.2% (*n* = 31) engaged in hobbies and 18.7% (*n* = 20) owned pets.

**TABLE 1:** Demographic and clinical characteristics of participants (*N* = 107).

Variables	*n*	%
**Age (years)**		
18–25	4	3.7
26–34	87	81.3
35+	16	15.0
**Gender**		
Male	19	17.8
Female	86	80.4
Non-binary	1	0.9
Prefer not to say	1	0.9
**Relationship status**		
Single	58	54.2
In a relationship	32	29.9
Married	1	0.9
Divorced and/or separated	16	15.0
**Duration of training (*n* = 106)**		
First year	50	47.2
Second year	49	46.2
Third+ year	7	6.6
**Current rotation**		
Anaesthesia	8	7.5
Family medicine	7	6.5
Internal medicine	26	24.3
Orthopaedics	14	13.1
Obstetrics and gynaecology	8	7.5
Psychiatry	14	13.1
Paediatrics	13	12.1
Surgery	17	15.9
**Active faith participation**		
Yes	73	68.2
No	34	31.8
**Engaged in hobbies (*n* = 106)**		
Yes	31	29.2
No	75	70.8
**Pet in the household**		
Yes	20	18.7
No	87	81.3

### Prevalence of burnout and perceived stress

Of the 107 participants, 97 (32%) completed the PSS and 78 (26%) completed the CBI. Among the 78 participants completing the full survey, the overall prevalence of burnout among participants was 79.4% (95% CI: 68.8–87.8). Among the subtypes of burnout, personal burnout was the most prevalent at 96% (95% CI: 89.2–99.2), followed by work-related burnout at 93.6% (95% CI: 85.7–97.9), while client-related burnout was lower at 30.7% (95% CI: 20.8–42.2). The prevalence of high perceived stress was 63.8% (95% CI: 53.5–74.3).

### Factors associated with burnout

Logistic regression analysis examined associations between burnout and demographic, lifestyle and perceived stress levels (*n* = 78, Table 2). High perceived stress was significantly associated with burnout, with interns experiencing high perceived stress being 5.3x more likely to report burnout (odds ratio [OR] = 5.30, 95% CI: 2.01–14.11, *p* < 0.01). Engaging in hobbies was associated with lower odds of burnout (OR = 0.16, 95% CI: 0.05–0.52, *p* < 0.01). Other variables did not show significant associations with burnout.

**TABLE 2:** Factors associated with burnout (*N* = 78).

Factors	Odds ratio	95% Confidence interval	*p*-value
**Gender**			
Male (reference)	1	-	-
Female	0.49	0.10–2.42	0.38
**Marital status**			
Single (reference)	1	-	-
Married	2.33	0.26–21.13	0.45
In a relationship	0.48	0.15–1.53	0.21
**Active faith participation**			
Yes (reference)	1	-	-
No	1.30	0.40–4.21	0.66
**Engaged in hobbies**			
No (reference)	1	-	-
Yes	0.16	0.05–0.52	0.002*
**Pet in the household**			
No (reference)	1	-	-
Yes	0.44	0.11–1.72	0.24
**Perceived stress**			
Low (reference)	1	-	-
High	5.3	1.99–14.11	0.001*

* , Denotes significance at *p* < 0.05.

### Factors associated with perceived social stress

Logistic regression analysis examined associations between perceived stress and demographic and lifestyle factors. The analysis showed that engagement in hobbies was associated with lower odds of high perceived stress (OR = 0.34, 95% CI: 0.13–0.84, *p* = 0.02) (Table 3). Other demographic and lifestyle variables did not show significant associations with perceived stress.

**TABLE 3:** Factors associated with high perceived stress (*N* = 78).

Factors	Odds ratio	95% Confidence interval	*p*-value
**Gender**			
Male (reference)	1	-	-
Female	0.76	0.26–2.23	0.62
**Marital status**			
Single (reference)	1	-	-
Married	1.89	0.53–6.71	0.32
In a relationship	1.37	0.52–3.62	0.52
**Age group (years)**			
18–25 (reference)	1	-	-
26–34	3.52	0.30–40.49	0.31
≥ 35	5.00	0.35–71.90	0.24
**Duration of training**			
First year (reference)	1	-	-
Second year	0.67	0.28–1.59	0.36
> Second year	0.91	0.15–5.52	0.92
**Active faith participation**			
Yes (reference)	1	-	-
No	1.59	0.63–3.98	0.32
**Hobbies**			
No (reference)	1	-	-
Yes	0.34	0.13–0.84	0.02*
**Pets**			
No (reference)	1	-	-
Yes	1.16	0.39–3.42	0.79

* , Denotes significance at *p* < 0.05.

### Multivariate analysis of burnout

In the multivariate logistic regression model (*n* = 78), high perceived stress remained an independent predictor of burnout (aOR = 1.28, 95% CI: 1.06–1.62, *p* = 0.02). Engagement in hobbies as an independent factor reduced the odds of burnout by 83% (aOR = 0.17, 95% CI: 0.06–0.71, *p* = 0.01). Further analysis of burnout subtypes (*n* = 78) indicated that perceived stress was significantly associated with personal burnout (OR = 4.89, 95% CI: 1.29–18.58, *p* = 0.02) and work-related burnout (OR = 3.46, 95% CI: 1.05–11.44, *p* = 0.04) but not client-related burnout (OR = 1.72, 95% CI: 0.68–4.37, *p* = 0.25).

## Discussion

This study aimed to investigate the prevalence of burnout and perceived stress among medical interns in Namibia as well as to explore associations between these variables and socio-demographic and lifestyle factors. The results indicated a high prevalence of overall burnout (79.4%), with personal burnout and work-related burnout more prevalent than client-related burnout. Furthermore, perceived stress emerged as a significant predictor of burnout, while interns who engaged in hobbies had significantly lower odds of burnout. These findings underscore a clear need for interventions to mitigate burnout and stress among Namibian medical interns.

The high prevalence of burnout found in this study is in keeping with global research on medical professionals, where rates have been reported to range from 37% to 76%.^6,10,11,12^ A recent study on community service doctors in South Africa reported burnout levels exceeding 80%.^34^ Similarly, another local study by Brückner et al.^15^ found that over half of medical doctors in a maternity unit were at risk of burnout, supporting a concerning trend of burnout in the Namibian healthcare system.

The predominance of personal and work-related burnout over client-related burnout is consistent with a single-site finding from Roslan et al.,^14^ who also reported higher personal and work-related burnout among medical trainees. The elevated burnout rates among Namibian interns reflect the demanding nature of the internship, including long working hours, high patient loads and limited institutional support, factors commonly reported in studies from other LMICs.^11,12,35^

The association between perceived stress and burnout found in this study further emphasises the critical role of stress as a precursor to burnout. Medical interns who reported high perceived stress were found to be five times more likely to report burnout. This finding supports that of Fisseha et al.,^11^ who demonstrated a threefold increase in emotional exhaustion among interns with high perceived stress levels. Stress is a well-established precursor to burnout,^2^ and these results underscore the need for stress management interventions aimed at Namibian interns to help prevent burnout.

The findings of this study also support the Job Demand-Resources Model, which suggests that high job demands (such as stress) can deplete personal resources (imbalance of situational and individual factors), leading to burnout.^2^ Given the demanding nature of medical internships, addressing stress is essential for preventing its harmful consequences. The findings of this study also align with the growing reports of dissatisfaction and distress among Namibian medical interns.^21^ The high prevalence of burnout observed in this cohort is likely linked to systemic challenges that may be difficult to address immediately and adequately. This further highlights the need for targeted interventions to support medical interns who are at the coalface of these challenges.

One of the findings of this study was that engagement in hobbies was a protective factor against developing burnout. While prior studies have largely focused on structural and occupational interventions, personal coping mechanisms such as hobbies have received less attention. This finding aligns with the literature suggesting that leisure activities can buffer stress and improve overall well-being.^36^ In addition, hobbies may serve as a form of stress relief, allowing interns to regain a sense of control and autonomy outside the clinical environment. Engagement in hobbies may reduce burnout by facilitating psychological detachment from work, promoting recovery from work-related stress and giving a sense of personal agency and identity outside the clinical role.^37^

Addressing burnout effectively requires targeting both individual and workplace factors. At the individual level, strategies such as engagement in hobbies, physical activity, mindfulness, time management, social support and sleep hygiene may promote recovery, emotional regulation and resilience, offering low-cost, immediately implementable interventions.^37^ At the workplace level, interventions are more challenging but crucial to tackle systemic drivers of burnout. These include reducing excessive working hours, ensuring adequate supervision, clarifying roles and responsibilities, promoting a supportive organisational culture while providing access to mental health services and balancing rotation design to prevent sustained high stress. While structural interventions require institutional buy-in and policy change, they directly address the root causes of work-related burnout, which was prominent in this cohort.

Although certain factors, such as pet ownership, active faith participation and relationship status, appeared to have potential associations with burnout, these relationships were not statistically significant. This suggests that while these factors may play a role in individual well-being, they may not exert a sufficiently strong effect to significantly influence burnout rates among medical interns. However, future research with larger sample sizes may provide more insight into these associations, especially as they pertain to coping strategies and support systems.

This study has several strengths, including its focus on an understudied population in an LMIC setting, its use of validated burnout and stress measures and its exploration of factors such as hobbies. However, certain limitations are acknowledged. The cross-sectional design limits causal inferences, highlighting the need for longitudinal studies to assess burnout over time. Furthermore, the study’s reliance on self-reported measures may introduce recall and response biases. Although survey completion varied, with 107 who completed the socio-demographic questionnaire, 97 the PSS and 78 the full survey, this was lower than the required sample size estimate (*n* = 161) and may affect generalisability. The high observed burnout prevalence suggests that key associations remain detectable. The sample size completing the full study was 78 (26%), and this voluntary response sampling could introduce selection bias as participants who choose to respond could differ from non-responders, further limiting the generalisability of the findings. Despite these limitations, this study provides insight into the burden of burnout among Namibian medical interns and highlights awareness before any consideration for intervention.

## Conclusion

The findings of this study reveal high levels of burnout and perceived stress in a limited sample of medical interns, with significant demographic and lifestyle association factors. The findings align with public media reports of distress and dissatisfaction in this occupational group. This raises both awareness of the impact and possible interventions promoting stress reduction, leisure activities and work-life balance as a means to addressing these challenges and interventions at an individual and organisational level.^38,39^
